# Combined predictive value of uric acid and serum lipid for stroke events in non-valvular atrial fibrillation patients

**DOI:** 10.3389/fcvm.2025.1569904

**Published:** 2025-03-26

**Authors:** Yuqi Tang, Baiqing Song, Tesfaldet H. Hidru, Yiheng Yang, Fei Liu, Jiatian Li, Chenglin Li, Yuhang Wen, Zhongzheng Yang, Ying Chen, Xiaolei Yang, Yunlong Xia

**Affiliations:** Department of Cardiology, First Affiliated Hospital of Dalian Medical University, Dalian, China

**Keywords:** non-valvular atrial fibrillation, stroke, serum uric acid, triglycerides, low-density lipoprotein

## Abstract

**Background:**

Serum uric acid (SUA) and lipid metabolism disorders are closely associated with atrial fibrillation (AF) and its prognosis. In patients with non-valvular AF (NAF), we evaluated the combined predictive value of SUA, triglycerides (TG), and low-density lipoprotein (LDL) for stroke to enhance stroke risk prediction and management.

**Methods and results:**

We included 3,176 NAF patients treated at the First Affiliated Hospital of Dalian Medical University from January 2020 to December 2023. We analyzed SUA concentration and lipid profile, along with relevant clinical data, to assess their impact on the occurrence of ischemic stroke (IS) in NAF patients. Due to gender differences in TG (1.39 mmol/L vs. 1.28 mmol/L for males, *P* = 0.031;1.57 mmol/L vs. 1.28 mmol/L for females, *P* = 0.001) and SUA levels (424 µmol/L vs. 397 µmol/L for males, *P* = 0.008; 361 µmol/L vs. 328 µmol/L for females, *P* = 0.004), we determined the thresholds for SUA (400 µmol/L in males and 330 µmol/L in females) and TG (1.28 mmol/L in males and 1.29 mmol/L in females) that predict stroke events in NAF patients by restricted cubic spline curves. Kaplan–Meier cumulative risk analysis indicates that a gender-based combined assessment of SUA and TG enhances stroke risk stratification in NAF patients. Compared to patients with low levels of SUA and TG, those with high levels of these biomarkers have a higher risk of IS (HR = 1.98). On multivariable Cox regression analysis with potential confounders, elevated SUA and low-density lipoprotein (LDL) levels were significantly associated with an increased risk of stroke. In summary, we developed the CHA_2_DS_2_-VASc+SUA+TG+LDL stroke risk prediction model. Its clinical predictive value was assessed using Harrell's C-statistic (C-index), integrated discrimination improvement (IDI) statistics, and net reclassification index (NRI) analysis.

**Conclusions:**

SUA, TG and LDL were strongly associated with stroke for NAF. The combination of SUA, TG, and LDL effectively enhanced the predictive value of the CHA_2_DS_2_-VASc score for IS.

## Introduction

Atrial fibrillation (AF) stands out as the most prevalent cardiac arrhythmia, affecting approximately 1.6% of Chinese adults, a figure that rises sharply with age (1, 2). Numerous epidemiological studies have flagged AF as a major contributor to ischemic stroke (IS) and overall cardiovascular mortality (3–5). This chaotic heart rhythm disrupts effective atrial contractions, leading to stagnation of blood flow and significantly raising the risk of thrombus formation in the left atrium. As a result, individuals with AF face a staggering fivefold increase in their risk of experiencing an IS compared to those without the condition (3, 6). Moreover, AF can worsen other health issues, including metabolic disorders (7, 8), hypertension, and diabetes, further elevating the threat of IS.

While uric acid is often associated with gout, its role extends far beyond that. Increasingly recognized as a biomarker for systemic inflammation and oxidative stress—two critical factors in the development of AF—uric acid has gained momentum for its implications in cardiovascular health. Studies reveal that hyperuricemia affects 20.7% of men and 5.6% of women, with these figures climbing annually (9). Recent research even highlights a potential connection between elevated serum uric acid (SUA) levels and subclinical AF (SCAF) (10). Furthermore, hyperuricemia has been reported to contribute to the recurrence of AF after catheter ablation (11). Suggesting it plays a key role in the persistence and re-emergence of this condition.

The Framingham study has shown that prolonged exposure to lipid imbalances can lead to atherosclerotic cardiovascular disease and increased mortality (12). Research from Luanluan Sun et al. indicates that higher plasma levels of the low density of lipoprotein (LDL) and triglycerides (TG) are directly linked to an elevated risk of IS, while showing a reduced risk of intracerebral hemorrhage (13, 14). Dyslipidemia also significantly impacts dementia in patients with AF (15). The link between AF and metabolic syndrome involves complex mechanisms, including activation of the sympathetic nervous system and impaired mitochondrial calcium management (16).

Dyslipidemia and hyperuricemia are common metabolic disorders that often go unnoticed for extended periods, leading to a lack of targeted clinical care and risk assessment guidelines (7, 8, 17, 18). While the CHA_2_DS_2_-VASc score is a well-established tool for evaluating stroke risk in NAF patients, it doesn't capture all individuals at high risk for thromboembolic events (19). To improve detection and enhance clinical decision-making, we have developed a new model: CHA_2_DS_2_-VASc+SUA+TG+LDL. This innovative approach aims to more effectively identify high-risk stroke populations among NAF patients, paving the way for better-targeted management and prevention strategies.

## Methods

### Study design

The study was conducted on patients diagnosed with NAF between January 1, 2020, and December 31, 2023. It aimed to assess the risk factors for stroke in patients with NAF. Briefly, this is an ancillary study designed to explore the impact of SUA, TG, and LDL on the prognosis of AF patients. Our study protocol was registered with the Chinese Clinical Trial Registry on September 21, 2020 (https://www.chictr.org.cn, registration number: Chi- CTR2000038377).

### Study population

**Figure 1:**
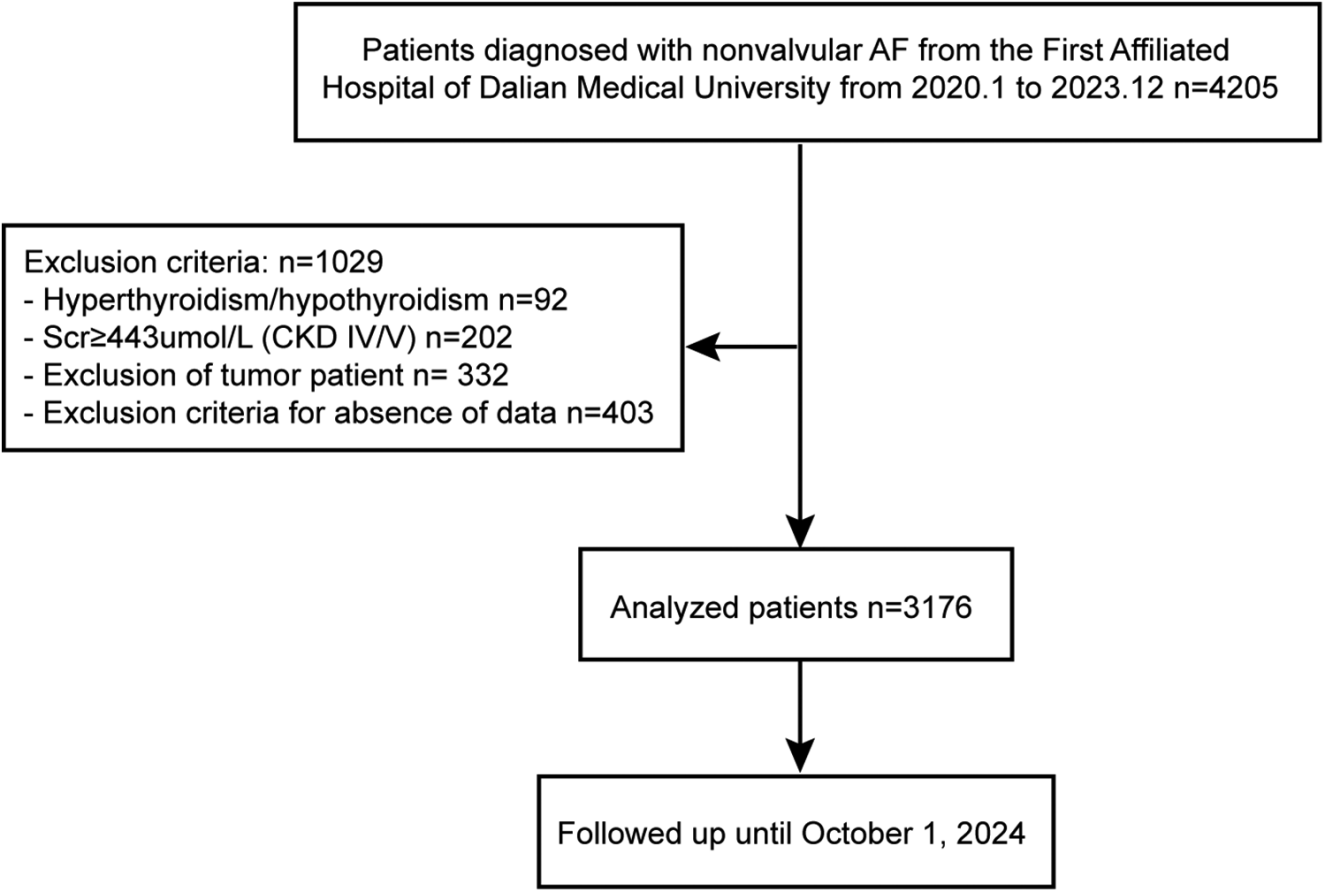
Flowchart of the included patients and follow-up. AF, atrial fibrillation; Scr, serum creatinine; CKD, chronic kidney diseases.

This study initially recruited 4,205 patients aged between 18 and 80 years with NAF. Patients with hyperthyroidism or hypothyroidism, serum creatinine (Scr) levels ≥443 µmol/L, and chronic kidney disease stages 4 or 5 (CKD IV/V) were excluded from the study. Also, patients with tumors and those missing key clinical covariates were excluded from the study. Ultimately, the final analysis included a total of 3,176 patients. Figure 1 provides an overview of the participant selection process. The research was conducted in accordance with the Declaration of Helsinki guidelines and received approval from the institutional review board of the First Affiliated Hospital of Dalian Medical University (FAHDMU). Informed consent was obtained from all participants, and all procedures described were performed in compliance with the approved guidelines.

### Baseline data and variables

Demographic and clinical characteristics, such as age, gender, and major risk factors for AF—including serum lipid levels, diabetes mellitus (DM), arterial hypertension, alcohol consumption, smoking, and other cardiovascular comorbidities—were obtained from electronic health records. All measurements, including serum concentrations of SUA, TG, total cholesterol (TC), and LDL cholesterol, were conducted at the FAHDMU laboratory following standard protocols.

### Definitions and follow-up

Follow-up was performed via database search and telephone call. The ischemic cerebrovascular events were recorded. IS includes all cardioembolic strokes —both definite and probable. We employed the Trial of ORG 10172 in Acute Stroke Treatment (TOAST) classification to differentiate stroke subtypes (20) with the aim of controlling selection bias. Diagnostic criteria for ischemic stroke include: (1) sudden onset of focal neurological deficits, (2) duration of ≥24 h or imaging evidence confirming ischemic injury to brain tissue, and (3) exclusion of cerebral hemorrhage through CT/MRI. All patients were followed up until the endpoint event occurred or December 31, 2023, whichever came first. The median follow-up time was 31 months (interquartile range, IQR 20–43 months).

### Statistical analysis

**Figure 2:**
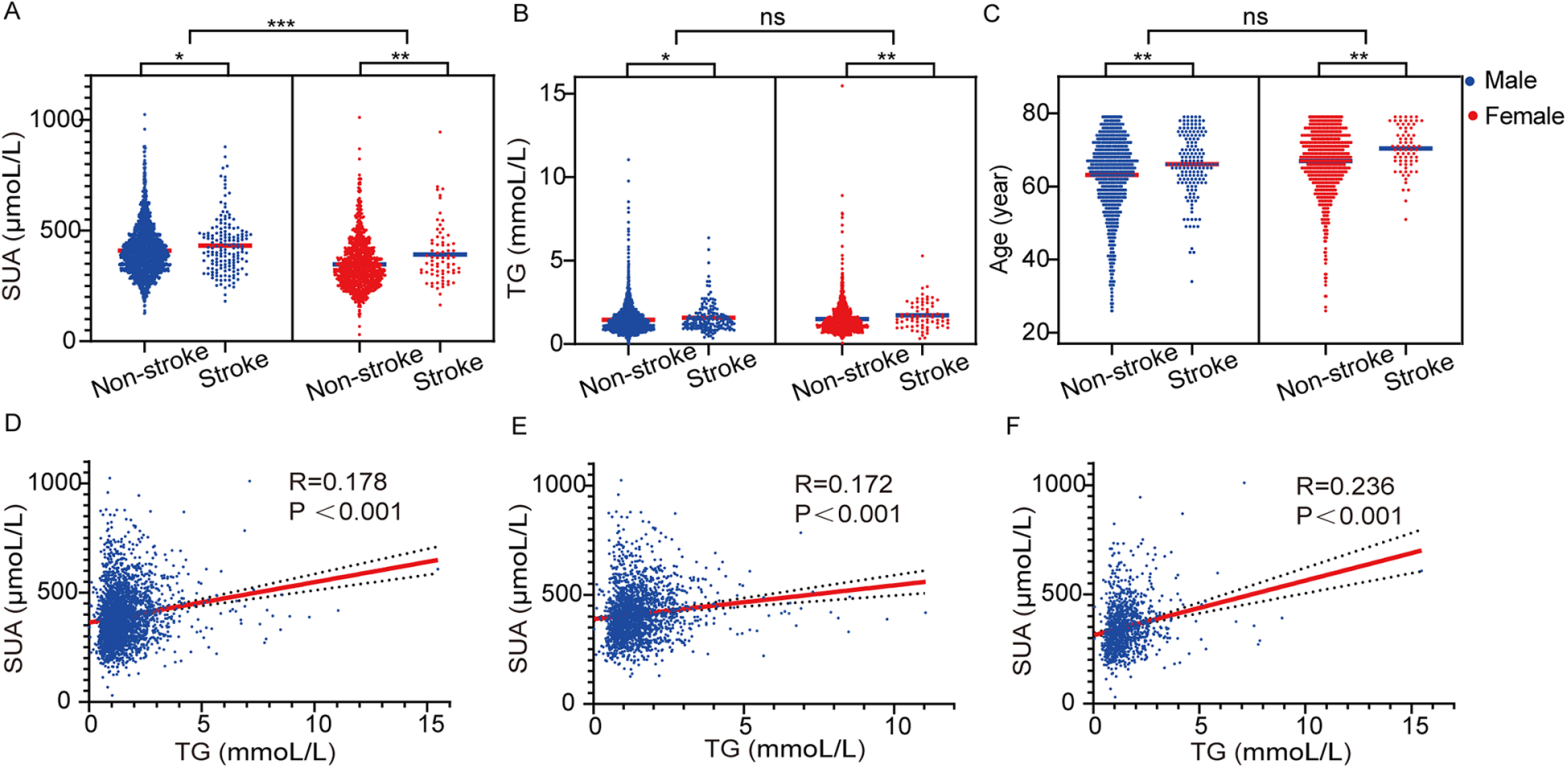
Serum uric acid **(A)**, TG **(B)** and age **(C)** in patients with or without stroke. The correlation of SUA and TG in total population **(D)**, males **(E)** and females **(F).**

The baseline characteristics were presented as the mean ± SD or interquartile ranges (IQR) for continuous variables, and categorical variables were reported as counts and percentages. Where appropriate, comparisons were made according to the Mann- Whitney *U* or Kruskal Wallis test. For categorical variables, differences were assessed using the χ^2^ or Fisher's exact test. Cox regression analyses were used to establish the relationship between the independent variables and stroke. Cox multivariate regression analyses were performed, adjusting for a pre-specified list of covariates identified based on significant indicators from the Cox univariate regression analyses. The results were presented as hazard ratios (HR) with 95% confidence intervals (CI). Given the gender differences in TG and SUA between the stroke and non-stroke groups (as shown in Table 1 and Figure 2), a restricted cubic spline analysis was conducted to quantitatively assess the threshold values for TG or SUA related to the occurrence of stroke events. The HR of 1 serves as the reference point; an HR greater than 1 indicates a higher risk of stroke, while an HR less than 1 suggests a lower risk. Kaplan–Meier curves were plotted to show the time to stroke based on SUA and TG levels among both stroke and non-stroke patients, with comparisons made using the log-rank test. To assess the discriminatory power for stroke incidence over 31 months, we evaluated several models: the CHA_2_DS_2_-VASc model, CHA_2_DS_2_-VASc+SUA model, CHA_2_DS_2_-VASc+SUA+TG model, and the CHA_2_DS_2_-VASc+SUA+TG+LDL model. The evaluation was assessed using receiver operating characteristics (ROC), C-index, NRI, and IDI. We employed calibration curves to evaluate the performance of the final model (CHA_2_DS_2_-VASc plus SUA, TG, and LDL).

**Table 1:** Baseline clinical characteristics of the study participants.

Variables	Male				Female			
	Total *n* = 2,081	Non-stroke *n* = 1,922	Stroke *n* = 159	*P*-value	Total *n* = 1,095	Non-stroke *n* = 1,023	Stroke *n* = 72	*P*-value
Age (year)	65 (58–71)	65 (58–71)	66 (62–73)	0.001	68 (63–73)	68 (63–73)	70 (67–76)	0.001
Smoking	586 (28)	541 (28)	45 (28)	0.967	125 (11)	121 (12)	4 (6)	0.154
Alcohol use	406 (20)	372 (19)	34 (21)	0.606	88 (8)	85 (8)	3 (4)	0.305
Persistent AF	1,002 (48)	922 (48)	80 (50)	0.595	409 (37)	380 (37)	29 (40)	0.570
Hypertension	423 (39)	384 (38)	39 (54)	<0.001	729 (35)	651 (34)	78 (49)	0.005
DM	155 (14)	136 (13)	19 (26)	0.014	318 (15)	283 (15)	35 (22)	0.002
HF	112 (10)	109 (11)	3 (4)	0.48	233 (11)	212 (11)	21 (13)	0.12
CAD	574 (28)	513 (27)	61 (38)	0.071	228 (21)	207 (20)	21 (29)	0.002
CKD	10 (1)	9 (1)	1 (1)	0.154	18 (1)	15 (1)	3 (2)	0.495
COPD	11 (1)	10 (1)	1 (1)	0.154	18 (1)	15 (1)	3 (2)	0.528
Previous TIA/Stroke	94 (9)	82 (8)	12 (17)	<0.001	224 (11)	181 (9)	43 (27)	0.011
Previous PE/DVT	12 (1)	12 (1)	0	0.291	14 (1)	12 (1)	2 (1)	1
TC (mg/dl)	4.27 (3.55–5.06)	4.27 (3.54–5.04)	4.42 (3.66–5.23)	0.102	4.58 (3.89–5.31)	4.57 (3.88–5.28)	4.81 (4.02–5.76)	0.113
TG (mg/dl)	1.28 (0.93–1.75)	1.28 (0.93–1.75)	1.39 (1.03–1.83)	0.031	4.58 (3.89–5.31)	1.28 (0.96–1.77)	1.57 (1.11–2.25)	0.001
HDL (mg/dl)	1.04 (0.89–1.22)	1.04 (0.89–1.22)	1 (0.83–1.21)	0.029	1.18 (0.99–1.4)	1.18 (0.99–1.4)	1.18 (0.94–1.4)	0.33
LDL (mg/dl)	2.37 (1.85–2.92)	2.36 (1.84–2.91)	2.47 (1.98–2.95)	0.113	2.48 (2–3)	2.48 (1.99–2.99)	2.67 (2.11–3.19)	0.164
SUA (mol/L)	398 (333–473)	397 (332–469)	424 (345–493)	0.008	330 (273–408)	328 (272–404)	361 (309–445)	0.004
BNP, µmol/L	238 (92–605)	236 (91–600)	277 (106–692)	0.099	249 (109–645)	248 (108–644)	306 (136–619)	0.357
PLT, ×10^9^/L	201 (170–240)	201 (170–239)	198 (173–254)	0.469	217 (184–259)	217 (183–259)	221 (189–261)	0.402
QTc (ms)	467 (444–490)	467 (444–490)	468 (447–491)	0.609	473 (450–498)	472 (450–497)	477 (450–500)	0.461
LAD (mm)	41 (38–46)	41 (38–46)	42 (39–46)	0.059	39 (36–43)	39 (36–43)	41 (37–45)	0.006
LVD (mm)	50 (47–54)	50 (47–54)	49 (47–54)	0.299	46 (43–49)	46 (43–49)	48 (44–50)	0.034
EF (%)	58 (53–59)	58 (54–59)	56 (52–59)	0.046	58 (55–59)	58 (55–59)	58 (55–59)	0.443
Anticoagulant	794 (73)	741 (72)	53 (74)	0.273	1,542 (74)	1,430 (74)	112 (70)	0.829
Anti-plate drug	293 (27)	270 (26)	23 (32)	0.016	611 (29)	551 (29)	60 (38)	0.304
ACEI/ARB	448 (41)	419 (41)	29 (40)	0.311	915 (44)	839 (44)	76 (48)	0.91
ARNI	238 (22)	221 (22)	17 (24)	0.224	531 (26)	484 (25)	47 (30)	0.69
β-blocker	652 (60)	611 (60)	41 (57)	0.599	1,189 (57)	1,095 (57)	94 (59)	0.642
CCB	255 (23)	234 (23)	21 (29)	0.122	461 (22)	418 (22)	43 (27)	0.222
Diuretic	361 (33)	334 (33)	27 (38)	0.021	704 (34)	637 (33)	67 (42)	0.397
Hypoglycemic agent	195 (18)	181 (18)	14 (19)	0.01	390 (19)	348 (18)	42 (26)	0.707
Ablation	150 (14)	137 (13)	13 (18)	0.266	276 (13)	255 (13)	21 (13)	0.983

Values are reported as median (interquartile range) or *n* (%). DM, diabetes mellitus; HF, heart failure; CAD, coronary artery disease; CKD, chronic kidney diseases; COPD, chronic obstructive pulmonary disease; TIA, transient ischemic attack; PE, pulmonary embolism; DVT, deep vein thrombosis; TC, total Cholesterol; TG, triglyceride; HDL, high-density lipoprotein; LDL, low-density lipoprotein; SUA, serum uric acid; BNP: brain natriuretic peptide; PLT: platelet; LAD, left atrial diameter; LVD, left ventricle diameter; EF, ejection fraction; ACEI, angiotensin-converting enzyme inhibitors; ARB, angiotensin-converting enzyme receptor blockers; ARNI, inhibitors of angiotensin receptor enkephalin peptidase; CCB, calcium channel blockers.

Furthermore, a decision curve analysis was performed to assess the clinical benefit of the final model. A two-sided *P*-value <0.05 was considered statistically significant. The statistical analyses were performed using SPSS software (version 26, IBM) and R software (version 4.4.2).

## Results

### Baseline characteristics

Between January 2020 and December 2023, a total of 3,176 patients were enrolled in the study. Among them, 231 patients had stroke attacks. Table 1 presents the baseline characteristics of the study population. Variables that revealed gender differences between stroke and non-stroke patients were age (66 vs. 65 years for males, 70 vs. 68 years for females), hypertension (34% vs. 37% for females, 45% vs. 41% for males), diabetes (39% vs. 38% for males, 49% vs. 34% for females), previous TIA/stroke (17% vs. 8% for males, 27% vs. 9% for females), TG levels (1.39 mmol/L vs. 1.28 mmol/L for males, 1.5 mmol/L vs. 1.28 mmol/L for females), and SUA levels (424 µmol/L vs. 397 µmol/L for males, 361 µmol/L vs. 328 µmol/L for females). The proportion of males using anti-platelet drugs, diuretics, and hypoglycemic agents was higher in the non-stroke group compared to the stroke group (all *P* < 0.05). Male stroke patients typically exhibited lower levels of HDL and reduced ejection fraction (EF). Strokes were more frequently observed in individuals with coronary artery disease (CAD, *P* = 0.002), as well as in those with larger measurements of left atrial diameter (LAD) and left ventricle diameter (LVD). In both men and women, there were no significant differences between stroke and non-stroke groups concerning smoking, alcohol use, persistent AF, heart failure (HF), CKD, chronic obstructive pulmonary disease (COPD), previous pulmonary embolism/deep vein thrombosis (PE/DVT), TC, LDL, QTc, anticoagulants, angiotensin-converting enzyme inhibitors angiotensin-converting enzyme receptor blockers (ACEI/ARB), inhibitors of angiotensin receptor enkephalin peptidase (ARNI), *β*-blockers, calcium channel blockers (CCB), and ablation treatments.

### The impact of SUA and TG levels on stroke

Given the observed differences in SUA, TG levels, and age in stroke events among males and females, we further analysed their impact on stroke occurrence. As illustrated in Figure 2, both SUA and TG levels, along with age, were significantly elevated in stroke patients compared to those without stroke (all *P* < 0.05). Importantly, irrespective of stroke status, men displayed higher SUA levels than women (Figure 2A). Additionally, we found that SUA and TG levels were significantly and positively correlated in the overall population, as well as within both male and female subgroups [*R* = 0.178, *P* < 0.001; *R* = 0.172, *P* < 0.001; and *R* = 0.236, *P* < 0.001, respectively (see Figures 2D–F)]. This suggests a potential mutual enhancement effect between these two metabolic biomarkers.

### Distribution of stroke-related risk factors

To evaluate the risk factors influencing stroke in NAF patients, we conducted a Cox regression analysis. In the univariate analysis, age, hypertension, DM, CAD, previous TIA stroke, LDL, TG, SUA, LAD, anti-plate drugs, CCBs, and diuretics were positively associated with an increased risk of stroke (Table 2). After adjusting for confounding variables, multivariable analysis revealed that age, previous TIA stroke, LDL, and SUA were independent predictors [with adjusted hazard ratio (HR) and confidence interval (CI) of 1.028 (1.011–1.044), 2.325 (1.674–3.23), 1.258 (1.058–1.495), 1.002 (1.001–1.003)] of stroke. This indicates that LDL could be regarded as a significant marker influencing stroke events in patients with NAF.

**Table 2:** Risk factors for stroke according to the Cox proportional hazards regression model in total population.

Variables	Univariate analysis			Multivariate analysis		
	Hazard ratio	95% CI	*P*	Hazard ratio	95% CI	*P*
Age (year)	1.037	1.021–1.021	<0.001	1.028	1.011–1.044	0.001
Gender (female)	1.199	0.908–1.584	0.201			
Smoking	0.93	0.679–1.276	0.654			
Alcohol use	1.019	0.717–1.448	0.919			
Hypertension	1.84	1.421–2.381	<0.001	1.185	0.878–1.6	0.268
DM	1.816	1.339–2.463	<0.001	1.204	0.858–1.691	0.283
Persistent	1.135	0.877–1.470	0.337			
CAD	1.713	1.308–2.243	<0.001	1.201	0.889–1.622	0.234
Previous TIA/Stroke	2.989	2.208–4.046	<0.001	2.325	1.674–3.23	<0.001
Previous PE/DVT	0.924	0.23–3.717	0.911			
HF	0.934	0.612–1.426	0.753			
COPD	1.853	0.69–1.98	0.221			
CKD	1.732	0.644–4.656	0.276			
LDL (mg/dl)	1.22	1.031–1.444	0.02	1.258	1.058–1.495	0.009
TC (mg/dl)	1.123	0.997–1.264	0.059			
TG (mg/dl)	1.12	1.011–1.24	0.03	1.052	0.938–1.179	0.388
HDL (mg/dl)	0.649	0.403–1.043	0.072			
SUA (mol/L)	1.002	1.001–1.003	<0.001	1.002	1.001–1.003	<0.001
QTc (ms)	1.002	0.999–1.005	0.206			
LAD (mm)	1.025	1.006–1.045	0.011	1.005	0.983–1.027	0.679
LVD (mm)	1.002	0.982–1.023	0.819			
EF (%)	0.993	0.978–1.007	0.325			
Anticoagulant	0.88	0.662–1.171	0.381			
Anti-plate drug	1.395	11.066–1.825	0.015	1.284	0.973–1.695	0.077
ACEI/ARB	1.072	0.827–1.389	0.598			
ARNI	1.19	0.892–1.587	0.238			
β-blocker	0.983	0.768–1.295	0.997			
CCB	1.337	1.002–1.783	0.048	1.262	0.943–1.689	0.117
Diuretic	1.351	1.039–1.756	0.025	1.093	0.831–1.438	0.524
Hypoglycemic agent	0.827	0.711–0.961	0.013	1.206	0.882–1.649	0.24
Ablation	1.138	0.791–1.638	0.486			

The multivariate analysis was adjusted for HBP, DM, CAD, previous TIA stroke, LDL, TG, SUA, LAD, anti-plate drug, CCB, diuretic, and hypoglycemic agent.

### Quantitative analysis of markers and their impact on stroke

**Figure 3:**
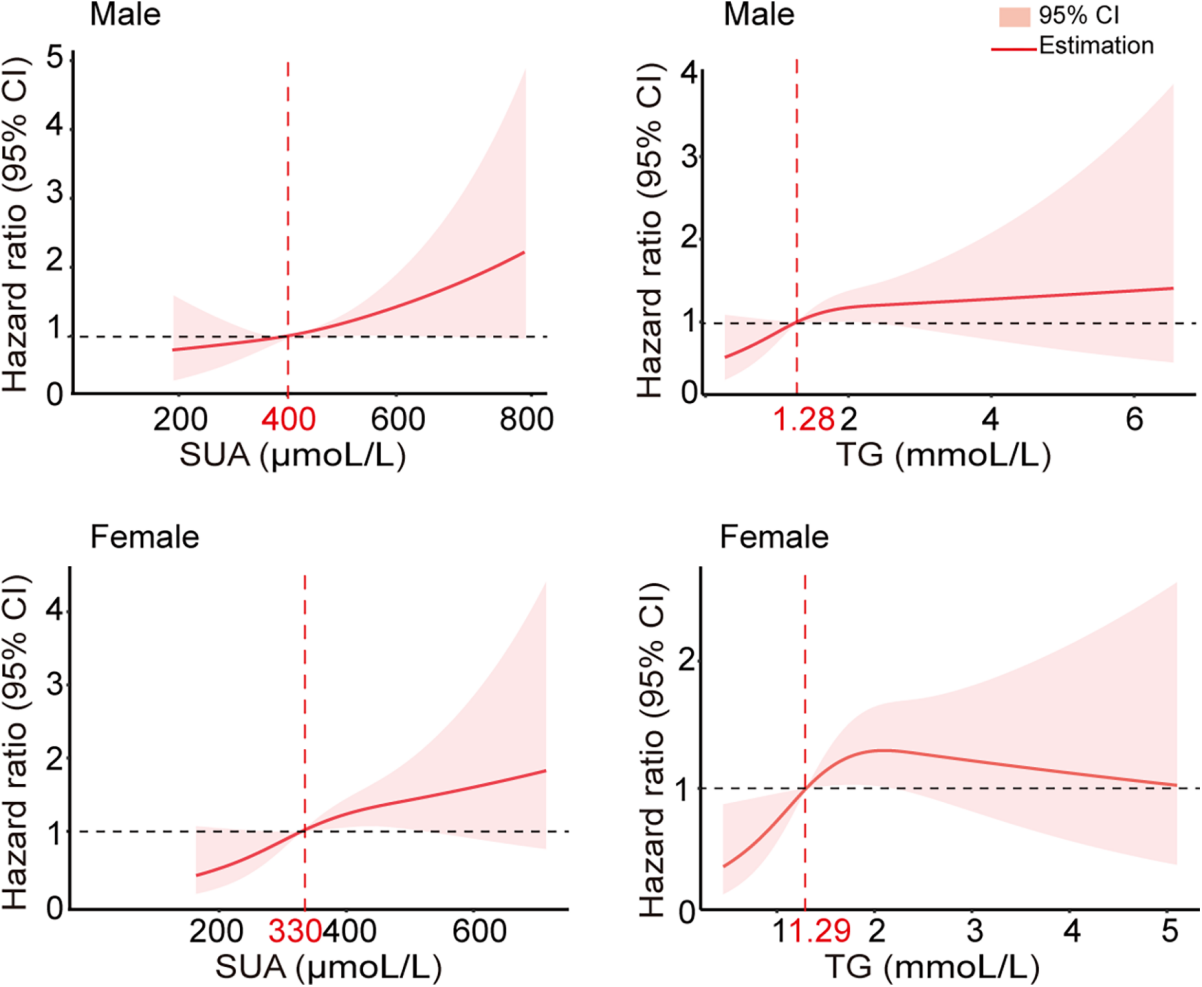
Multivariable adjusted hazard ratios for stroke incidence according to levels of SUA and TG on a continuous scale. Red lines are multivariable-adjusted hazard ratios, with pink areas showing 95% CIs derived from restricted cubic spline regressions with three knots at the 25th, 50th, and 75th percentiles. The dashed grey lines indicate reference lines for no association at a hazard ratio of 1.0. Analyses were adjusted for age, previous TIA stroke, SUA, and TG.

**Figure 4:**
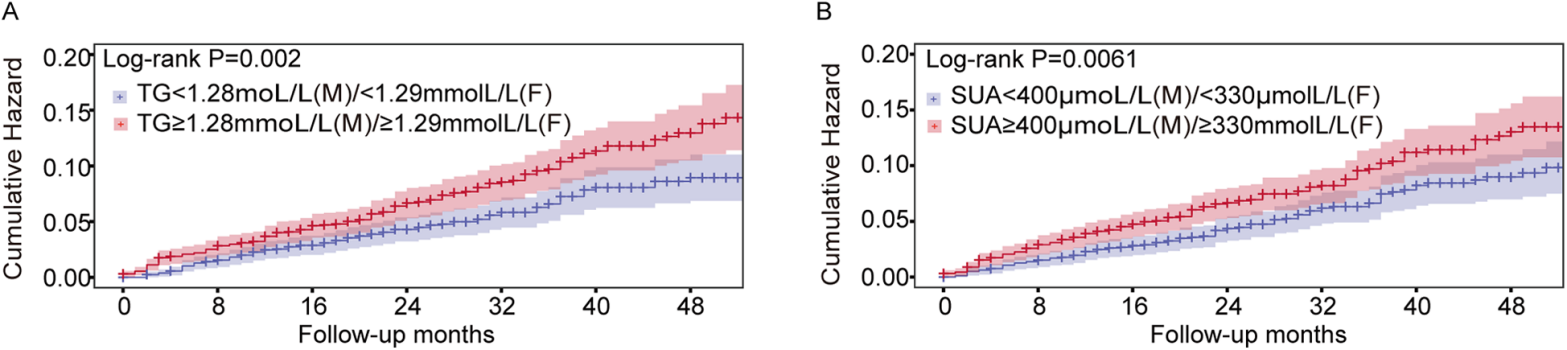
Kaplan–Meier curves showing the incidence of stroke for different levels of SUA and TG. **(A)** A Kaplan–Meier survival curves for TG, the cut-off points for TG 1.28 mmol/L in males and 1.29 mmol/L in females, respectively. **(B)** Kaplan–Meier survival curves for SUA; the cut points for SUA were 400 µmol/L in males and 330 µmol/L in females, respectively.

**Figure 5:**
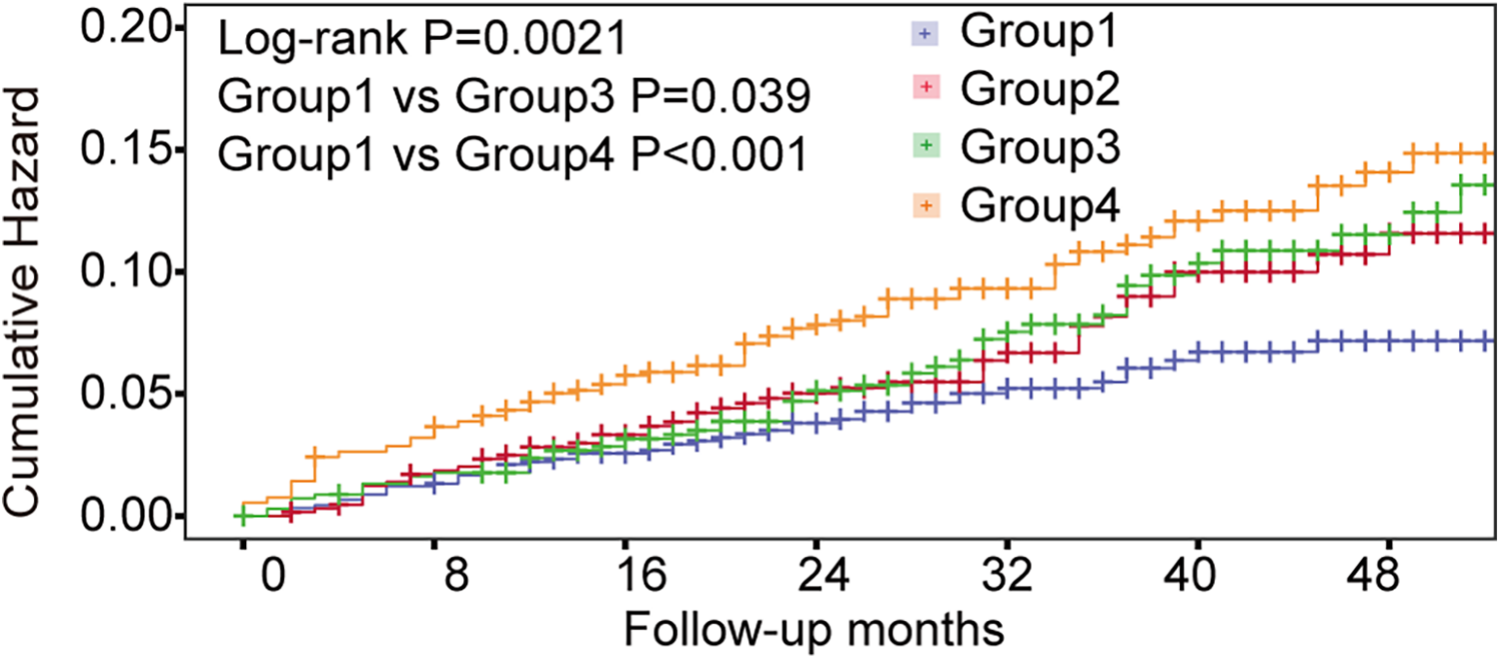
Kaplan–Meier curve showing the incidence of stroke. All patients were divided into 4 categories. Group 1: patients with both low-level SUA and TG (blue); Group 2: patients with high-level SUA and low-level TG (red); Group 3: patients with high-level TG and low-level SUA (green); Group 4: patients with high levels of both TG and SUA (orange).

To determine the risk thresholds for increased stroke events associated with SUA and TG levels, we conducted a quantitative analysis employing restricted cubic spline curves for both SUA and TG (see Figure 3). The SUA and TG values corresponding to an HR of 1 were used as thresholds to distinguish between low-risk and high-risk groups. Specifically, the cut-off values were established at 400 µmol/L for SUA in males and 330 µmol/L in females, while for TG, the thresholds were set at 1.28 mmol/L for males and 1.29 mmol/L for females. To assess the effectiveness of these threshold values for risk stratification, we plotted Kaplan–Meier curves. The analysis revealed that individuals with elevated TG levels faced a significantly increased cumulative hazard of stroke events (Figure 4A, HR = 1.509, *P* = 0.002). Similar results were observed for SUA levels (Figure 4B, HR = 1.439, *P* = 0.0061). Stroke risk varied according to SUA and TG levels, as depicted in Figure 5. Based on the low- and high-risk categories defined by the spline curves for SUA and TG, we classified all patients into four groups: Group 1 included patients with low levels of both SUA and TG (blue); Group 2 consisted patients with high SUA and low TG levels (red); Group 3 comprised patients with high TG and low SUA levels (green); and Group 4 encompassed those with high levels of both TG and SUA (orange). Notably, patients in Group 4 exhibited a higher incidence of stroke (log-rank test, *P* = 0.0021). The HRs for patients in Groups 2, 3, and 4 were 1.435, 1.514, and 1.98, respectively. In summary, these findings indicate that the cut-off values effectively distinguish between lower-risk and higher-risk groups, with the combined effect of SUA and TG demonstrating an even more pronounced impact.

### The additive effect of SUA, TG and LDL levels on the CHA_2_DS_2_-VASc model

**Figure 6:**
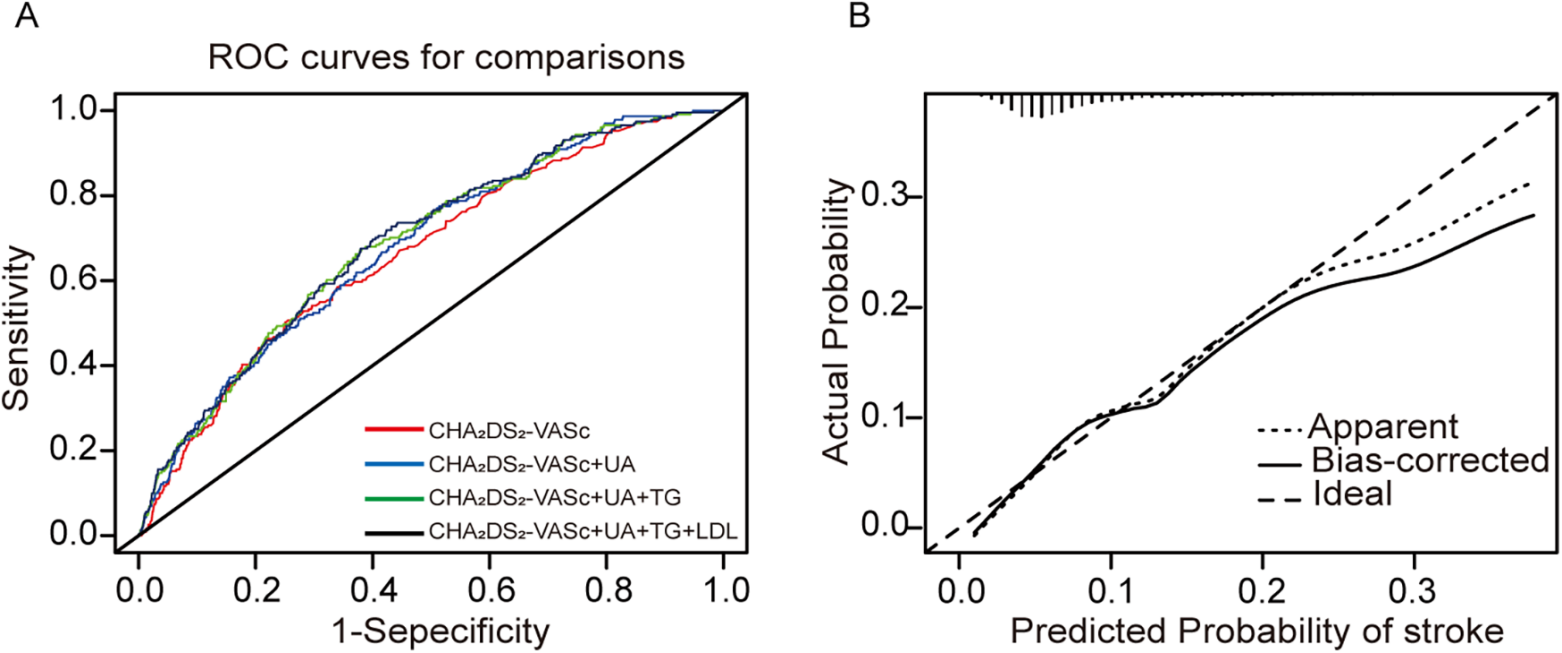
Model-comparison results of predicting the 31-month incidence of IS. **(A)** Receiver operating characteristics (ROC) curves of freedom from stroke at 31 months for the different models. **(B)** Calibration curves for the final model (CHA_2_DS_2_-VASc+SUA+TG+LDL). The dotted line represents the entire cohort and the solid line is the result after bias correction by bootstrapping (1,000 repetitions), indicating model performance (boot mean absolute error = 0.007).

Considering the variations in biomarkers SUA, TG, and LDL between stroke and non-stroke patients, we have developed new stroke assessment models, referred to as model 2: CHA_2_DS_2_-VASc+SUA, model 3: CHA_2_DS_2_-VASc+SUA+TG, model 4: CHA_2_DS_2_-VASc+SUA+TG+LDL. To evaluate the diagnostic utility of these models compared to CHA_2_DS_2_-VASc (model 1), we performed ROC analysis (Figure 6A). The area under the curve (AUC) for the models were as follows: model 1: 0.664 (95% CI: 0.628–0.664), model 2: 0.678 (95% CI: 0.644–0.678), model 3: 0.687 (95% CI: 0.653–0.687), and model 4: 0.689 (95% CI: 0.655–0.689). Notably, the clinical diagnostic utility of model 4 is more pronounced (*P* = 0.042, Table 3).

**Table 3:** Comparison of different risk prediction models.

Variables	Model 1	Model 2	Model 3	Model 4
AUC (95% CI)	0.664 (0.628–0.664)	0.678 (0.644–0.678)	0.687 (0.653–0.687)	0.689 (0.655–0.689)
*P*-value	Ref	0.169	0.061	0.042
C-index	0.664 (−0.418–1.746)	0.678 (−0.44–1.796)	0.682 (−0.565–1.93)	0.689 (−0.662–2.041)
NRI (95% CI)	Ref	0.032 (−0.013–0.087)	0.038(−0.006–0.098)	0.065 (0.003–0.011)
*P*-value	–	0.189	0.143	0.02
IDI (95% CI)	Ref	0.007 (0.003–0.01)	0.008 (0.004–0.013)	0.011 (0.006–0.017)
*P*-value	–	0.001	<0.001	<0.001

Comparison of various risk prediction models using area under the curve (AUC), Harrel's C-statistic (C-index), integrated discrimination improvement (IDI) statistics, net reclassification index (NRI), and their corresponding 95% confidence intervals (CIs).

**Figure 7:**
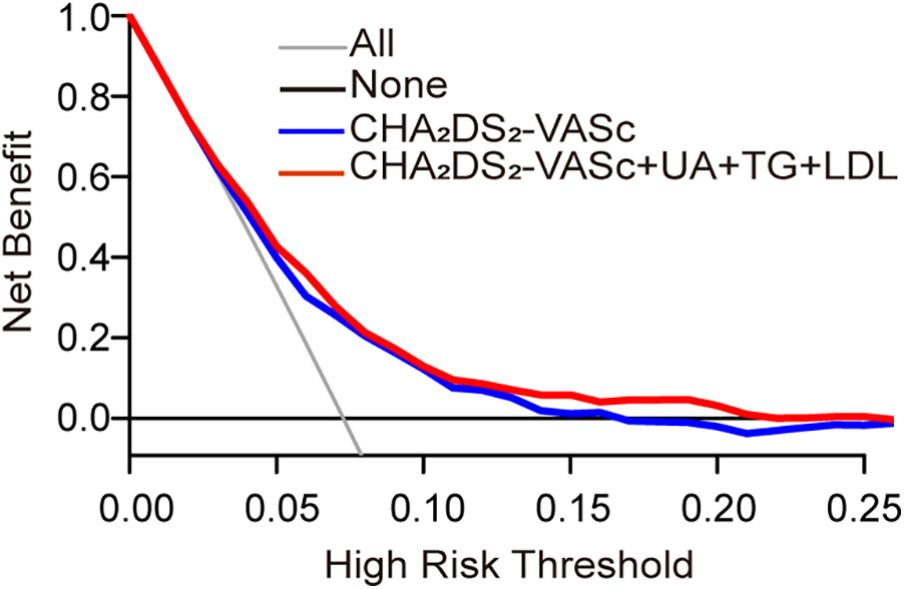
Decision curve analyses (DCA) of the CHA_2_DS_2_-VASc model and final model for 31 months of stroke incidence. The *x*-axis indicates the threshold incidence for stroke at 31 months, and the *y*-axis indicates the net benefit. The horizontal dark grey line assumes no patients will experience the event; the light grey line assumes all patients will experience the event. The final model had enhanced net benefit compared with the CHA_2_DS_2_-VASc model at risk threshold >2.6%.

To assess the accuracy and reliability of the predictive model 4 by C-index, IDI, and NRI. Model 4 achieved the highest C-index (0.689), indicating greater predictive capability. Model 4 improved classification ability by approximately 6.48% compared to model 1, including SUA, TG, and LDL, contributing to this enhanced predictive capability (*P* = 0.02). The IDI analysis further demonstrated that model 4 improved predictive ability by 1.1% compared to model 1 (*P* < 0.001). These findings suggest that model 4 significantly enhances predictive ability and offers more accurate stroke risk predictions. Furthermore, calibration curves illustrated a strong concordance between predicted probabilities and the observed incidence of stroke events (Figure 6B). The decision curves for model 1 and model 4 indicate that the net benefit of model 4 surpasses the threshold probabilities ranging from 0.0 to 0.26 (Figure 7).

## Discussion

In this study, the incidence of ischemic stroke was 7.3%. Additionally, SUA, LDL, age, and previous TIA stroke were independently associated with IS in patients with NAF. Notably, a linear correlation was observed between TG and SUA. By integrating SUA, TG, and LDL with the CH_2_DS_2_-VASc score, the ability to identify stroke risk in NAF patients was significantly improved.

**Figure 8:**
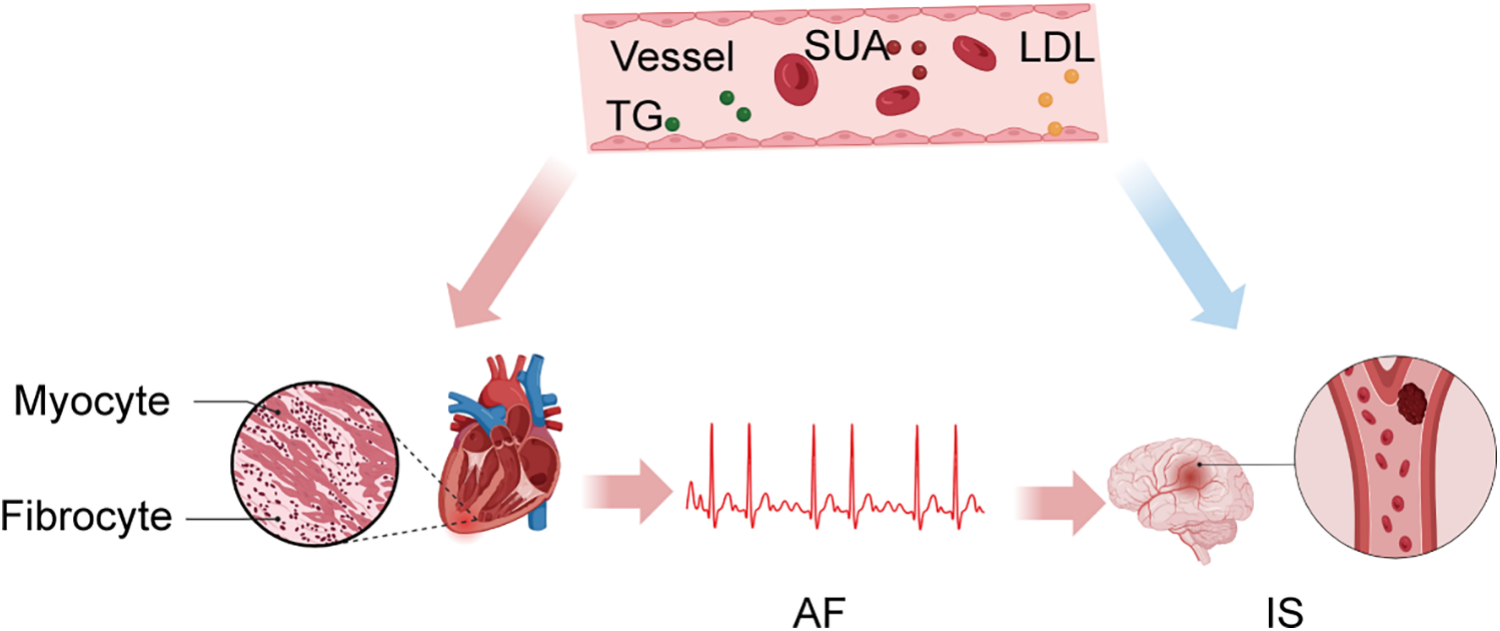
SUA, TG, and LDL promote and maintain AF through inflammation while acting directly on intracranial vessels, leading to IS.

The relationship between SUA levels and AF reveals a fascinating interplay of pathophysiological mechanisms. Elevated SUA in the bloodstream, a condition known as hyperuricemia, is more than just a metabolic concern—it can lead to endothelial dysfunction, a critical precursor to atherosclerosis, often recognized as a significant risk factor for AF. But that's not all; uric acid also contributes to oxidative stress by generating free radicals, which can inflict damage on myocardial tissue. This disruption in heart function may pave the way for arrhythmias. Moreover, the inflammatory response is a major player in AF development, and uric acid is a known activator of inflammation (Figure 8). By triggering the NLRP3 inflammasome, it promotes the release of pro-inflammatory cytokines, like interleukin-1β (IL-1β), which can lead to structural changes in the atria and further exacerbate AF (21).

Recent clinical studies underscore the urgency of this issue, demonstrating that individuals with elevated SUA levels face a significantly higher risk of developing AF compared to those within normal ranges. Notably, a large prospective cohort study indicated that hyperuricemia correlates with an increased incidence of AF and its related complications, hinting that elevated uric acid may act as a valuable prognostic marker (22). Elevated levels of SUA are believed to induce oxidative stress and inflammatory responses, which can lead to endothelial dysfunction and increase the risk of atherosclerosis, thereby heightening the likelihood of stroke (23, 24). Uric acid may directly damage endothelial cells, promoting thrombosis (25). Additionally, the uric acid to creatinine ratio has emerged as a noteworthy predictor for the recurrence of AF post-ablation (26).

In our study, we found that stroke patients exhibited elevated levels of SUA and TG, regardless of gender. The specific thresholds identified for increased stroke risk differ between men and women, indicating distinct pathophysiological mechanisms and underscoring the need for tailored approaches in clinical assessment and management. These findings are consistent with prior studies that suggest these patients tend to be older (23, 27–30). Our study revealed higher critical SUA thresholds for men (400 µmol/L) compared to women (330 µmol/L), which may be attributed to hormonal differences. Estrogen may enhance uric acid excretion in women, allowing men to accumulate more SUA before experiencing an increased stroke risk, thereby highlighting men's greater vulnerability to hyperuricemic conditions at elevated SUA levels.

Regarding triglycerides, the cutoff level shows only minor gender differences: 1.28 mmol/L for men and 1.29 mmol/L for women. This slight variation may relate to lipid metabolism changes influenced by sex hormones, smoking, and unhealthy lifestyles, which affect triglyceride synthesis and clearance, subsequently altering the lipid profile and stroke risk factors by gender. These findings emphasize the importance of exploring how gender differences in SUA and TG impact stroke risk stratification. Acknowledging these gender-specific physiological and hormonal dynamics is essential in developing individualized risk profiles. Estrogen not only facilitates uric acid excretion but also influences lipid metabolism, leading to distinctive cardiovascular risk patterns in women compared to men. Thus, a universal approach may be inadequate in stroke risk assessment. Incorporating gender-specific thresholds and risk factors into existing models could result in more precise and effective clinical strategies.

Cumulative risk analysis involving SUA and TG in the NAF population reveals an intriguing insight: elevated levels of these biomarkers not only raise stroke risk when considered individually, but when both are simultaneously elevated, the risk amplifies dramatically. This echoes the findings from earlier studies that explored the roles of SUA and homocysteine in detecting SCAF (10). In the past, a comprehensive nationwide cohort study further supported that the likelihood of clinical events increases progressively with the rise of TC and TG levels, with stroke risk comprising a striking 10% of these occurrences (14) (Figure 8). Elevated levels of TG can lead to increased plasma viscosity and hemodynamic changes, thereby enhancing the risk of thrombosis (27, 28). Additionally, TG metabolites may promote inflammatory responses in endothelial cells, exacerbating atherosclerosis (31–33). Adding another layer to this narrative, research examining the triglyceride-glucose index unveils interesting gender disparities in TG levels (34). Thus, incorporating SUA and TG into our risk assessment model seems logical and valuable. However, we recognize that further clinical studies are essential to better understand the relationship between TG and AF.

A retrospective cohort study sheds light on the connection between metabolic syndrome and atrial remodeling (35). Notably, LDL plays a pivotal role among the components of metabolic syndrome, significantly contributing to both atherosclerosis and its correlation with AF (36, 37). A prospective cohort study strengthens this association by demonstrating that higher LDL levels are linked to an increased risk of AF recurrence post-catheter ablation (38). Consistent with these observations, our findings indicate that elevated LDL levels correlate independently with heightened stroke risk in patients with NAF, even after controlling for potential confounders, yielding a hazard ratio of 1.25 (95% CI 1.058–1.495, *P* = 0.009). The established significance of LDL in cardiovascular risk cannot be overstated; prior research suggests that lowering LDL levels by just 1 mmol/L with statins can reduce ischemic stroke risk by 20% (39, 40). LDL is often oxidized, which promotes the formation of atherosclerosis. Oxidized LDL triggers inflammatory and immune responses (41), leading to endothelial dysfunction and potentially causing instability in atherosclerotic plaques (42, 43), which may increase the risk of stroke in NAF patients (44, 45). The above evidence underscores the importance of managing cholesterol levels in stroke prevention strategies. However, it is essential to adopt a balanced approach to cholesterol management, as excessively low cholesterol levels in the bloodstream may increase the permeability of vascular walls (46).

Management of AF primarily involves anticoagulation therapy to prevent stroke. Moreover, effective heart rate and rhythm control are crucial for comprehensive AF management in addition to anticoagulation. Despite these interventions, predicting individual stroke risk and managing the potential bleeding complications associated with anticoagulation therapy remain challenging. The CHA_2_DS_2_-VASc score, widely used for stroke risk stratification, considers variables such as congestive heart failure, hypertension, age, diabetes, prior stroke, vascular disease, and sex category. Since its introduction by the European Society of Cardiology in 2010 for stroke prevention in non-valvular AF, the landscape of patient management has shifted significantly, expanding beyond mere prevention to include the management of associated comorbidities and risk factors like obesity. In the current study, we have integrated SUA and lipid markers (TG and LDL) with the CHA_2_DS_2_-VASc model to refine stroke risk prediction further. This integration aims to enhance predictive accuracy and improve patient-specific management strategies.

SUA has emerged as the second most prevalent metabolic disorder, yet its management—alongside lipid levels—often receives insufficient attention in patients with AF. When it comes to preventing AF, maintaining a BMI within the healthy range of 20–25 kg/m^2^ is strongly recommended (47). In the past, research has underscored the influence of SUA and lipid markers in promoting atrial fibrosis through inflammation, which not only supports the persistence of AF but also increases the risk of stroke [ ]. Our study highlights the importance of addressing both SUA and lipid levels. Promisingly, our findings demonstrate that the CHA_2_DS_2_VASc+SUA+TG+LDL model significantly improves predictive capabilities regarding stroke risk. This enhanced model exhibits superior performance metrics—such as AUC, C-index, NRI, and IDI—compared to the traditional CHA_2_DS_2_-VASc model. In essence, integrating SUA and lipid markers into our predictive model not only elevates its efficacy but also advances our efforts toward more accurate stroke risk assessments.

## Study limitations

Firstly, the study's design as a single-center retrospective analysis inherently limits its generalizability. By concentrating on a specific population from a single medical facility, the findings may not be applicable to broader and more diverse populations. Additionally, the relatively small sample size further constrains the statistical power of the study. With a limited number of participants, there is a heightened risk of statistical anomalies, which could lead to findings that are skewed or not representative of the larger population. Moreover, our study does not delve into the mechanisms by which SUA and lipid markers, particularly TG and low-density lipoprotein LDL, may contribute to IS. Therefore, further research is imperative to examine the lower threshold levels of these markers and their direct mechanisms of action concerning the vascular damage associated with IS.

## Conclusions

In summary, our findings revealed a notable correlation between SUA, TG, and LDL levels and the incidence of IS events in patients with NAF. Interestingly, SUA and LDL emerged as key independent risk factors for IS in this patient group. By combining SUA, TG, and LDL with the CHA_2_DS_2_-VASc score, we found a significant boost in the accuracy of stroke predictions for those with NAF. This enhanced model could support clinicians in making more informed decisions.

## Data Availability

The raw data supporting the conclusions of this article will be made available by the authors, without undue reservation.
